# 肺癌循环肿瘤细胞的研究进展

**DOI:** 10.3779/j.issn.1009-3419.2012.10.09

**Published:** 2012-10-20

**Authors:** 正 王

**Affiliations:** 518020 深圳，暨南大学第二临床医学院深圳市人民医院胸外科 Department of Thoracic Surgery, Shenzhen People's Hospital, the Second Clinical Medicine College of Jinan University, Shenzhen 518020, China

**Keywords:** 肺肿瘤, 循环肿瘤细胞, 转移, 复发, Lung neoplasms, Circulating tumor cells (CTCs), Metastasis, Recurrence

## Abstract

转移和复发是肺癌患者死亡的主要原因。研究发现循环肿瘤细胞（circulating tumor cells, CTCs）在肺癌转移和复发中起着重要作用。而且随着靶向治疗的不断进步，对于晚期无法取得肺癌实体组织的患者，CTCs作为一种肺癌组织替代物可以决定治疗方案。所以CTCs在早期发现肺癌患者的微转移、检测肿瘤复发、评估预后和选择个体化治疗方案方面有着重要作用。本文针对CTCs的研究进展及肺癌领域的应用进行综述。

## Abstract (en)

Metastatic and recurrent tumors have been identified as the leading attribute to the lung cancer deaths. Cancer research has demonstrated the critical role circulating tumor cells (CTCs) play in the metastatic spread of carcinomas and the recurrence of lung cancer. The rapid advancement of technology in targeted therapy resolves the embarrassing situation for those late-stage patients whose tumor tissues cannot be obtained. CTCs, as a substitute for the tumor tissues, represent a decisive tool to the cancer treatment strategy. Thus, CTCs exert a fundamental role in the early detection of micro-metastasis, assisting in diagnosis, prognosis and monitoring of the recurrent tumors, and subsequently choosing an individualized approach for the therapeutic treatment. This article will review the advances, which have been made in the research area of CTCs with the aid of its applications in cancer therapy.

根据世界卫生组织最近公布的数据，肺癌已成为目前发病率和死亡率增长最快，对人类健康威胁最大的恶性肿瘤。在我国近几年来随着城市空气污染的加重，特别是PM2.5含量持续维持高位，肺癌的发病率和死亡率明显上升^[1]^。估计到2025年我国肺癌年死亡人数可达100万，将成为肺癌第一大国。随着个体化治疗的不断深入，表皮生长因子受体（epidermal growth factor receptor, EGFR）等基因突变检测和动态观察成为临床制定治疗方案和预测预后的重要依据。2011年美国国立综合癌症网络（National Comprehensive Cancer Network, NCCN）指南明确指出，对于肺腺癌、大细胞肺癌和组织学类型不明确的非小细胞肺癌（non-small cell lung cancer, NSCLC）等推荐检测*EGFR*突变类型。但是由于肺癌早期发现率低，约70%-80%的患者确诊时已为中晚期而无法取得肺癌组织标本，也无法接受基因和病理检测^[2]^。对于早期病人组织标本获取方法复杂，而且肺癌基因表型及分子表型可能在治疗过程中发生改变，出现药物耐药^[3]^。目前肺癌组织的分子学检测达不到实时、动态监测的目的。为了提高肺癌患者的生存率，降低死亡率，优选临床治疗方案，亟需一种手段不仅可以早期预测肺癌转移和复发，而且可以实时和动态监测肺癌相关基因的突变类型。

## 1 循环肿瘤细胞（circulating tumor cells, CTCs）概述

1869年Ashworth^[4]^在1例因癌症死亡的患者外周血中发现与原发肿瘤细胞体积和形态相似的细胞，从此提出了CTCs的概念。目前CTCs定义为自发或因诊疗操作脱离实体瘤原发灶或转移灶进入外周血循环的一类肿瘤细胞，其极少见于正常人^[5]^。经过免疫逃避未被清除的CTCs不仅在一定条件下可以发展为转移灶^[6]^，而且可以通过迁移、粘附、相互聚集形成循环微癌栓（circulating tumor microemboli, CTM）^[7]^。最新研究^[8]^表明这种微小癌栓还可以附着于血管内皮，导致血栓形成，引起静脉血栓栓塞（venous thromboembolism, VTE）。所以CTCs的检测与肿瘤转移、复发和预后评估有密切关系，而且对于晚期无法取得肿瘤组织的患者起源于实体瘤的CTCs为其靶向治疗的分子学检测开启了一扇大门。Maheswaran等^[9]^利用CTCs检测*EGFR*突变类型，其与原发肿瘤吻合率高达92%，而且CTCs来自于外周血，可以动态取样，这充分说明CTCs检测有可能作为一种基因检测手段，用于实时动态检测肿瘤患者的基因突变类型，指导临床个体化治疗。

## 2 CTCs检测方法

### 2.1 CTCs富集技术

由于CTCs在外周血存在数量极少（1个CTC/10^6^-10^7^单个核淋巴细胞）^[10]^，对于检测方法灵敏性要求很高，由于生物技术的局限性，研究者首先尝试将CTCs进行富集，提高其浓度，从而达到检测的目的。

密度梯度离心技术是在单个核淋巴细胞和CTCs的密度与其它血液细胞的密度不同的基础上，依靠淋巴细胞分离液进行梯度离心富集单个核淋巴细胞和CTCs，从而达到提高CTCs浓度的目的。在此技术上建立的免疫梯度离心技术（RosetteSep^TM^）是建立在阴性选择的基础上，利用单抗混合物使非靶细胞与全血中的多个红细胞交联，增加非靶细胞的密度，再通过密度梯度离心，去除非靶细胞，从而增加了CTCs富集的效率^[11]^。2000年Vona等^[12]^提出ISET（isolation by size epithelial tumor cells）技术，主要是利用肿瘤细胞与正常细胞大小不同来分离CTCs。血液通过孔径8 μm的滤膜过滤，使CTCs聚集于滤膜上，达到富集作用。Oncoquick离心法是在滤膜原理基础上，利用一个内置多孔屏障的专用50 mL试管进行密度梯度离心，避免了全血各个层面的交叉污染，检出率明显高于其它方法^[13]^。此类富集技术简单、方便，可以普遍使用。适合富集任何类型肿瘤的CTCs，富集的CTCs可以继续后续免疫学和基因检测。但由于需要血液标本量大，而且容易出现交叉污染和CTCs的丢失，限制了其进一步应用。

免疫学富集技术是目前应用最广泛的CTCs富集方法。其利用上皮源性的恶性肿瘤均表达上皮粘附因子（epithelial cell adhesion molecule, EpCAM）和细胞角蛋白（cytokeratins, CK）等标记物的特点，与连有免疫磁性微球（immunomagnetic microsphere, IMMS）的特异性单抗相结合，于外加磁场的作用下吸附复合物，达到富集靶细胞的作用^[14]^。Tewes等^[15]^利用体外和体内试验对比免疫磁珠技术与Oncoquick技术和RosetteSep^TM^技术，发现免疫磁珠技术回收CTCs的重复性和敏感性均高于其余两种技术。目前通过不同磁珠和外加磁场，利用阴性或阳性选择方式建立的免疫磁珠富集方法包括MACS系统、Dynabeads系统、EasySep系统等^[16-18]^。此技术优点为可以保持CTCs完整性，具有较高的重复性和敏感性。但是由于CTCs的异质性，可能导致CTCs遗漏。研究者^[14]^尝试联合其它的生物标记物富集CTCs，结果表明能明显提高富集率，而后述的CTCs鉴定技术也大多采用联合多种生物标记物检测的策略。

### 2.2 CTCs鉴别技术

随着生物技术的进步，越来越多的技术可以直接标记检测、鉴定CTCs的存在和数量，根据检测原理可分为两大类，即细胞计数法和核酸检测法。

#### 2.2.1 细胞计数法

流式细胞技术（flow cytometry, FCM）是目前最常用的细胞检测技术。其通过检测荧光特异性抗体与肿瘤细胞特异性抗原（CK和EpCAM）结合后产生的荧光信号来鉴别CTCs。由于CK和EpCAM细胞在部分的血细胞中也有表达^[19]^，所以Kerbs等^[20]^利用CD45标记血细胞，通过阴性选择将混杂的血细胞排除，达到提高特异性的目的。研究者利用生物标记组合，通过不同的光学检测设备设计了不同的检测系统，包括FAST系统、LSC系统、ACIS系统和ARIOL系统等。该方法最大的优点是定量检测CTCs的数量，可以对CTCs多种参数（大小形态、细胞类型、存活状态、细胞内外标记物、基因突变检测）进行分析，但检测灵敏性较低，需要的血液标本较多。

#### 2.2.2 核酸检测法

逆转录聚合酶链反应（reverse transcription polymerase chain reaction, RT-PCR）是目前最常用的核酸检测方法，由于特异性的mRNA通常不在正常外周血细胞中表达，而且外周血中存在RNA酶，原发肿瘤组织分泌mRNA进入血液中都被降解。因此我们可以推测外周血中检测到这些特异性mRNA全部来自于CTCs，间接提示CTCs的存在^[21]^。而定量RT-PCR（RT-qPCR）技术和多重RT-qPCR技术是在原有的基础上通过增加内参基因，利用靶基因的组合提高CTCs检测的特异性^[22]^。该方法的优点是具有高特异性、定量、快速，而且需要的血液较少。但是值得注意的是由于mRNA易降解、扩增效率和非特异性扩增的影响，会出现假性结果，该方法也破坏细胞的形态和功能，中断了继续分析的可能^[22-24]^。

### 2.3 富集和鉴定结合的技术

随着对CTCs研究的不断深入，单个技术检测CTCs的敏感性和特异性已不能满足研究者需求，研究者开始尝试利用富集技术和鉴定技术相结合的方法，提高CTCs检测的敏感性和特异性。

AdnaTest系统^[25]^通过免疫磁珠技术将EpCAM标记的CTCs进行富集，然后通过RT-PCR检测肿瘤细胞特异性表达的mRNA，达到检测CTCs的目的，该方法具有高特异性，便于发现和鉴别恶性肿瘤的特异性靶标。但是方法较为复杂，而且无法摆脱核酸检测技术的缺陷，破坏了细胞结构，无法进行后续研究。

CellSearch系统是目前唯一通过美国食品和药物管理局（Food and Drug Administration, FDA）批准用于临床的CTCs检测技术，已被应用预测转移性乳腺癌、结直肠癌和前列腺癌患者的无进展生存率（progression free survival, PFS）和总生存率（overall survival, OS），其富集方法与AdnaTest系统相似。首先利用免疫磁珠技术将EpCAM标记的CTCs从血液样本中富集出来，然后通过特殊的生物标记或化学染色（CK8/18/19和DAPI阳性、CD45阴性）准确识别CTCs^[25, 26]^。Balic等^[27]^利用CellSearch技术和Oncoquick技术同时检测61个肿瘤转移患者的CTCs，发现CellSearch技术无论敏感性和回收率都高于Oncoquick技术。CellSearch技术具有高敏感性和特异性，由于形成了固定的系统，并经过FDA认证，具有自动化、高效性、高重复性的特点，而且所需血液样本量少，易于重复检测；缺点为仍存在漏诊和误诊的情况，且检测费用较高，使其在临床上广泛应用受限，而且通过系统处理的CTCs回收困难，阻碍了CTCs的后续分析^[26, 27]^。为了进一步提高CTCs检测效率，Hou等^[28]^将ISET技术和CellSearch技术结合使用，辅以核染色的方法，不仅准确检测出CTCs，还证明了CTM的存在，但仍无法摆脱临床应用的局限性。

### 2.4 其它检测方法

2007年马萨诸塞州总医院开发了第一代CTC芯片（CTC-Chip），其在微流体学的CTCs检测技术基础上，采用微流芯片的技术，利用包被抗体的位点丛捕获流过芯片的CTCs，达到检测CTCs的目的。Nagrath等^[29]^利用CTC-Chip技术，通过EpCAM抗体筛选CTCs，筛选出的细胞再通过CK/DAPI/CD45来验证，发现该技术具有高敏感性和特异性。但是这种芯片不仅制造相对困难、成本昂贵，且显微位点周围的血流通畅性限制了与抗体覆盖位点接触的CTCs数量。而第二代CTC-Chip芯片（microvortex-generating herringbone-chip, HB-Chip）通过一种小室流动槽，使样品快速混合，增加了血液的通过量和效率；并通过安装标准载玻片，利用标准的病理检测方法对癌细胞进行鉴别，提高检测的准确性，捕获的CTCs还可用于其它检测或培养。研究证实HB芯片在CTC芯片的基础上提高了25%的癌细胞捕获率，可捕捉血液样本中超过90%的癌细胞，研究中从几个患者的血液样本中捕捉出4到12个循环肿瘤细胞群组成的CTM，其可能成为研究和检测CTM的新方法。CTC-Chip技术通过模拟生物微环境筛选CTCs，保持CTCs活性，具有高敏感性和特异性，可以继续后续的形态学和分子学的检测，并且需要的血液标本较少，易于重复测量。由于仍局限于EpCAM筛选CTCs，可能会产生假性结果^[30]^。

研究者^[31]^还利用酶联免疫斑点法（enzyme linked immunospot assay, ELISPOT）来检测乳腺癌和前列腺癌患者血液中CTCs的存在，通过检测CTCs分泌的相关蛋白-上皮特异性粘蛋白和前列腺特异性抗原，判断CTCs存在。此技术可以验证CTCs的活性，还能检测到肿瘤形成过程中细胞表达分泌的重要蛋白，对于CTCs与肿瘤转移复发的相关性研究具有重要意义。但是该方法检测灵敏度较低，而且目前无法估计CTCs的数量，应用于CTCs检测的可行性还需要进一步研究证实。

## 3 肺癌CTCs临床应用

目前普通的血液学检测和高分辨率的影像学检测仍然无法准确的诊断和预测肺癌患者转移、复发和预后，也无法实时、动态选择个体化治疗方案，而与转移和复发有密切关系的CTCs恰恰弥补了这些不足。

### 3.1 临床诊断和分期

由于血液系统是肿瘤转移的重要途径，CTCs存在逐渐成为诊断恶性肿瘤的潜在标准。研究^[32]^显示CTCs对肺癌诊断也具有潜在的应用价值。Bevilacqua等^[33]^利用CellSearch系统在1例肺组织穿刺活检诊断为低分化神经内分泌瘤的患者外周血中检测到EpCAM和CK阳性的CTCs，然而EpCAM通常在低分化神经内分泌瘤中不表达，最后在肝脏转移灶中确诊为EpCAM和CK阳性的小细胞肺癌（small cell lung cancer, SCLC）。Tanaka等^[34]^利用CellSearch技术比较125例肺癌患者和25例肺良性肿瘤患者的CTCs阳性率，其以1个CTC/7.5 mL作为分界点，发现肺癌患者阳性率（30.6%）明显高于良性肿瘤患者阳性率（12.0%），说明CTCs对于肺癌诊断具有指导意义，可能成为高危患者早期筛查协助诊治的重要手段。与传统诊断手段相比，CTCs在肺癌的诊断中还处于摸索阶段，还需要更多临床证据支持，但联合CTCs检查后的综合诊断会提高临床医师诊疗的警惕性。

虽然血液中检测到肿瘤细胞并不意味着一定存在转移灶，但研究^[35]^认为CTCs与肿瘤转移有密切关系，CTCs计数越多患者面临远处转移的负荷越重，未来发生转移的病灶数也越多。Tanaka等^[34]^利用受试者工作特征曲线（receiver operating characteristic curve, ROC曲线）证明了肺癌患者外周血中CTCs的含量跟肺癌进展特别是肺癌转移具有相关性。Hou等^[36]^的研究同样表明，CTCs的存在与肿瘤分期存在明显的相关性，CTCs计数随着疾病分期的增加而明显增加，广泛期患者计数明显高于局限期；而且发现SCLC患者外周血中CTCs计数明显高于其它类型的肺癌，这也验证了临床上SCLC患者更容易发生全身转移。Yie等^[37]^应用RT-PCR对143例NSCLC患者进行检测，发现表达Survivn mRNA的CTCs与肿瘤侵犯程度和淋巴结转移状态有关。目前CTCs可作为肺癌组织学类型及临床分期的有益补充，其存在可被视为肿瘤TNM分期的中M分期的真实状态。结合病理学和影像学的检查结果，可以更准确的反映患者当前病情。

### 3.2 预后判断和疗效评价

肺癌患者的预后和抗肿瘤药物的疗效与多种因素有关，目前众多的研究提示CTCs可早期判断预后，评价抗肿瘤药物疗效。一项小规模的临床研究^[38]^中利用CellSearch技术，以2个CTCs/7.5 mL作为分界点，发现复发肺癌患者比新发肺癌患者CTCs阳性率高（83% *vs* 78%），说明了CTCs与肺癌复发有密切关系。而且在其中12例患者的随访中同种方案一线化疗2个疗程后影像学变化与CTCs变化具有相关性，影像学中肿瘤好转伴随着CTCs计数的减少。虽然为小样本的临床实验，但是可以看到CTCs在肺癌的预后评估和疗效评价方面的重要性。

在NSCLC领域Hofman等^[39, 40]^认为利用ISET技术从手术后NSCLC患者外周血中分离出的细胞为非血细胞的循环细胞（circulating non-haematological cells, CNHCs），通过对比发现上述患者10 mL外周血中含有50个CNHCs以上患者的OS和PFS明显低于另一组，并且在Ⅰ期+Ⅱ期患者中具有较高的复发和死亡的风险。而CNHCs中CTCs的存在可能是导致阳性结果的重要原因。2011年Krebs等^[20]^利用CellSearch技术检测101例Ⅲ期-Ⅳ期NSCLC患者CTCs数量，未化疗前以5个CTCs/7.5 mL为分界点，发现其OS存在统计学差异（> 5：4.3个月； < 5：8.1个月）；同种方案化疗1周期后以2个CTCs/7.5 mL为分界点，多因素分析发现CTCs数量跟化疗后疗效和OS有关，说明CTCs检测在预测NSCLC患者化疗疗效和预后方面具有重要作用。

在SCLC中研究者利用CellSearch技术发现在50例患者中80%的患者CTCs阳性，其中在7.5 mL血液中CTCs含量 > 300的患者中位生存期（134天）明显低于CTCs含量 < 2的患者中位生存期（443天），而且经过1个疗程一线化疗后大多数患者出现CTCs含量下降，在22天时阳性率只有60%，说明CTCs可以反映SCLC变化和评估化疗方案疗效^[36]^。Hou等^[28]^在小样本研究（97例SCLC患者）中发现未治疗前CTCs数量和化疗后CTCs变化量可以预测患者OS。而且他们认为ISET技术和CellSearch技术筛选出的CTCs中还存在CTM和已凋亡的CTCs，他们通核染色方式将筛选出的CTCs分为单个有活性CTCs、CTM和凋亡CTCs三组，而在CTM中并未发现凋亡CTCs，这可能预示着CTM存在特殊的保护机制防止CTCs凋亡和化疗药物的杀伤，其可能成为肺癌治疗的新靶点。

在乳腺癌领域2012年即将公布初步结果的SWOG S0500试验中，随机选取500例（≥5CTCs）乳腺癌转移女性患者，研究其在化疗3周后CTCs持续高表达（≥5CTCs）换用其他化疗方案患者是否受益。其研究的主要终点为PFS和OS。利用这项实验结果将可以明确CTCs临床检测和治疗方案更换的意义，而这也可以预测在肺癌领域CTCs对于治疗方案变更的价值。

目前CTCs对于肺癌预后和疗效判断的研究样本量较少，而且肺癌CTCs的检测方法和使用的肿瘤标记物也不同，所以关于CTCs与肺癌预后关系的研究尚需通过大规模、多中心的随访研究进行验证。

### 3.3 个体化治疗应用

近年来基因诊断指导肿瘤个体化治疗的研究越来越受重视，由于晚期或非手术肿瘤患者组织标本获取困难，使基因检测在一定程度上受到限制，而且研究^[41]^发现由于肿瘤的异质性，肿瘤的演变过程中存在进化，原发部位和转移灶可能存在基因型的改变。因此，在肿瘤个体化治疗过程中实施动态监测原发灶和转移灶中肿瘤细胞及其基因变化成为亟需解决的问题。CTCs作为两者之间的桥梁，能同时反映原发灶和转移灶的性质，在个体化治疗中起重要作用。

*EGFR*的突变可以预测肺癌对于TKI靶向药物的疗效，在肺癌的个体化治疗中起着重要作用^[42]^。由于CTCs检测技术的局限，研究者首先想到利用血液循环中游离的肿瘤循环DNA进行检测肺癌*EGFR*突变。Kimura等^[43]^使用扩增阻滞突变系统（amplified refractory mutation system, ARMS）的方法检测了NSCLC患者的肿瘤组织和血清的*EGFR*突变信息并进行对比，结果发现具有较高的一致率（72.7%），这可能由于血清中游离DNA主要来自CTCs的凋亡所造成的。随着CTCs检测技术的不断进步，研究者开始利用CTCs自身来进行EGFR突变检测。Maheswaran等^[9]^利用CTC-Chip技术将NSCLC患者血液中的CTCs收集并提取DNA，再利用ARMS法检测其*EGFR*突变类型，其与原发肿瘤突变吻合率高达92%，明显高于血浆游离DNA突变吻合率。而且在肺癌患者CTCs的基因检测中发现20号外显子T790M位点突变与TKI药物耐药有明显关系，并且明显降低了患者PFS。这充分说明CTCs的检测可以在个体化治疗的过程中动态的监测肿瘤细胞及其*EGFR*基因变化。

随着靶向基因的不断涌现，肺癌CTCs也可用于其他生物标记物的检测，优化个体化治疗方案。最近的研究^[44]^中，研究者利用FAST系统检测晚期NSCLC患者CTCs中核苷酸切除修复交叉互补组1（excision repair cross complement group 1, ERCC1）的表达，发现ERCC1在NSCLC患者CTCs表达水平与铂类药物化疗疗效和生存期呈负相关，提示CTCs可用于预测铂类化疗的疗效和患者生存期。研究者^[36]^还应用CellSearch系统检测了SCLC患者外周血中的CTCs，并分析了死亡细胞血清标记物。结果提示在局限期及广泛期SCLC患者外周血均可检出CTCs，并通过检测CTCs中CD56证实其为肿瘤来源，提示CTCs及细胞死亡血清学标记物有可能成为促进药物开发的药效学标记。

CTCs对肺癌个体化治疗具有潜在重大意义，可以实时、动态的检测肺癌患者的分子学类型，反映肿瘤的生物学状态，为患者个体化治疗提供理论基础和临床证据。但能否完全代替原发组织标本指导分子靶向治疗，尚需大规模的前瞻性研究来证实。

## 4 困惑与障碍

自从CTCs发现以来人们不断研究其在肿瘤转移和复发中的作用，Fidler等^[45]^采用放射性碘标记肿瘤细胞注入动物体内，24 h后只有0.1%的肿瘤细胞保持存活，而形成转移的肿瘤细胞不到0.01%。为了解释肿瘤转移效率低这一现象，近年逐渐形成上皮-间充质转化（epithelial-mesenchymal transition, EMT）理论。EMT最初定义为在胚胎时期上皮细胞丧失其上皮特性而获得间充质细胞特性的过程，其在胚胎干细胞分化过程中起着关键作用^[46]^。研究发现在肿瘤患者中随着肿瘤细胞不断增殖，部分细胞也会出现EMT，导致上皮表型标志E-钙粘素表达下降，而波形蛋白、成纤维细胞特异性蛋白-1等间充质表型特征分子上调，致使细胞失去极性，粘附能力下降，运动能力增强，造成肿瘤细胞脱落于局部，一部分由于参与细胞结构组成的上皮细胞结构蛋白的缺失，致使细胞结构松弛变形，然后侵入结缔组织间隙并穿过基底膜进入血液循环系统，另一部分细胞由于肿瘤扩张生长，毛细血管破裂，直接进入血液系统，形成CTCs^[47]^。这就说明了肿瘤CTCs可能存在异质性，需要更多的生物标记物来检测。而极少数逃过免疫反应和凋亡的CTCs通过间充质-上皮转化（mesenchymal-epithelial transition, MET）重新获得上皮表型或者通过形成CTM在毛细血管末端形成癌栓，突破血管屏障，从而形成转移灶。De Wever等^[48]^研究发现CTCs检测困难可能是由于EMT过程中形成间质性CTCs，其在肿瘤转移过程中起着重要作用。Iwatsuki等^[49]^利用肿瘤患者外周血中CTCs找出于5个与EMT相关的基因。这不仅可以作为一种新型检测CTCs的基因靶点，而且也进一步验证了EMT在CTCs形成过程中的重要性。除此之外肿瘤干细胞等理论的存在，都表明仅仅依靠上皮细胞特异的生物标记物或者单一的生物标记物并不能代表肿瘤CTCs的全部特征。还需要更多关于肿瘤复发、转移的分子和遗传机制的深入研究，以达到提高检测技术的特异性和敏感性的目的，使其应用于临床。

## 5 展望

综上所述外周血CTCs检测在肺癌转移的早期诊断和预后预测方面具有重要意义，其可以指导分子靶向药物的应用、评估治疗疗效。虽然外周血CTCs检测方法进步迅速，但仍有以下问题值得注意：①具有高特异性和敏感性的技术仍是应用于临床检测的关键，临床检测CTCs的技术仍要改善；②更多、更特异的生物标记物，特别是基于EMT、肿瘤干细胞和CTM等CTCs理论相关的生物标记物的发现，有助于提高肺癌CTCs检测的准确性和重复性；③进一步深化肺癌复发和转移的分子和遗传机制研究，将有助于提高CTCs检测的预测性和指导性，并有可能成为新抗肺癌药物开发和筛选的基础；④在兼顾准确性的同时也要选择尽量简单、易行、花费小的方法，将有助于CTCs尽快的进入临床。CTCs的检测和研究将有助于提高肺癌患者的生活质量和生存时间，必将成为临床肺癌诊治的必要手段。
